# Serum Proteomic Analysis Identifies SAA1, FGA, SAP, and CETP as New Biomarkers for Eosinophilic Granulomatosis With Polyangiitis

**DOI:** 10.3389/fimmu.2022.866035

**Published:** 2022-06-10

**Authors:** Jing Xiao, Shaohua Lu, Xufei Wang, Mengdi Liang, Cong Dong, Xiaoxian Zhang, Minzhi Qiu, Changxing Ou, Xiaoyin Zeng, Yanting Lan, Longbo Hu, Long Tan, Tao Peng, Qingling Zhang, Fei Long

**Affiliations:** ^1^ Sino-French Hoffmann Institute, State Key Laboratory of Respiratory Disease, School of Basic Medical Sciences, Guangzhou Medical University, Guangzhou, China; ^2^ Pulmonary and Critical Care Medicine, Guangzhou Institute of Respiratory Health, National Clinical Research Center for Respiratory Disease, National Center for Respiratory Medicine, State Key Laboratory of Respiratory Diseases, The First Affiliated Hospital of Guangzhou Medical University, Guangzhou, China; ^3^ Health Management Center, Shenzhen People’s Hospital (The Second Clinical Medical College, Jinan University; the First Affiliated Hospital, Southern University of Science and Technology), Shenzhen, China; ^4^ Guangdong South China Vaccine Co., Ltd, Guangzhou, China

**Keywords:** eosinophilic granulomatosis with polyangiitis, biomarkers, severe asthma, data-independent acquisition, parallel reaction monitoring

## Abstract

**Background:**

Eosinophilic granulomatosis with polyangiitis (EGPA) is characterized by asthma-like attacks in its early stage, which is easily misdiagnosed as severe asthma. Therefore, new biomarkers for the early diagnosis of EGPA are needed, especially for differentiating the diagnosis of asthma.

**Objectives:**

To identify serum biomarkers that can be used for early diagnosis of EGPA and to distinguish EGPA from severe asthma.

**Method:**

Data-independent acquisition (DIA) analysis was performed to identify 45 healthy controls (HC), severe asthma (S-A), and EGPA patients in a cohort to screen biomarkers for early diagnosis of EGPA and to differentiate asthma diagnosis. Subsequently, parallel reaction monitoring (PRM) analysis was applied to a validation cohort of 71 HC, S-A, and EGPA patients.

**Result:**

Four candidate biomarkers were identified from DIA and PRM analysis—i.e., serum amyloid A1 (SAA1), fibrinogen-α (FGA), and serum amyloid P component (SAP)—and were upregulated in the EGPA group, while cholesteryl ester transfer protein (CETP) was downregulated in the EGPA group compared with the S-A group. Receiver operating characteristics analysis shows that, as biomarkers for early diagnosis of EGPA, the combination of SAA1, FGA, and SAP has an area under the curve (AUC) of 0.947, a sensitivity of 82.35%, and a specificity of 100%. The combination of SAA1, FGA, SAP, and CETP as biomarkers for differential diagnosis of asthma had an AUC of 0.921, a sensitivity of 78.13%, and a specificity of 100%, which were all larger than single markers. Moreover, SAA1, FGA, and SAP were positively and CETP was negatively correlated with eosinophil count.

**Conclusion:**

DIA-PRM combined analysis screened and validated four previously unexplored but potentially useful biomarkers for early diagnosis of EGPA and differential diagnosis of asthma.

## Introduction

Eosinophilic granulomatosis with polyangiitis (EGPA), formerly named Churg-Strauss syndrome, is a rare multisystemic disease characterized by wheezing symptoms, granulomatous, eosinophilia (EOS)-rich inflammation, and systemic necrotizing vasculitis, affecting small-to-medium size blood vessels (1). EGPA is a puzzling disease that combines asthmatic manifestation with hypereosinophilic syndromes and anti-neutrophil cytoplasmic antibody (ANCA)-associated vasculitis features (2). EGPA has three clinical and histological stages—allergic stage composed of asthma and sinusitis, an eosinophilic stage characterized by peripheral hypereosinophilia and intra-organ infiltration of eosinophils, and the final is vasculitis stage including necrotizing inflammation of small vessels and end-organ damages (3). Among them, wheezing symptoms are the main clinical manifestation of EGPA, similar to asthma, especially severe asthma, which is considered to be one of the predominant diseases in the initial phase of EGPA (4). However, not all EGPA patients have such successive phases, as clinical manifestations may vary widely.

Currently, the most popular classification criteria for EGPA are the 1990 American College of Rheumatology (ACR) classification criteria, including 1) asthma, 2) paranasal sinus abnormality, 3) peripheral blood EOS (>10%), 4) unfixed pulmonary infiltration, 5) mononeuropathy or polyneuropathy, and 6) extravascular EOS on history. Four out of six criteria should be present in an EGPA patient (5). However, these criteria are not diagnostic, as their goal is to classify patients as having a probable diagnosis of EGPA, but only once vasculitis has already been diagnosed (6). Therefore, the diagnosis of EGPA is still clinically based on a complementary investigation. In addition, the current clinical classifying of EGPA into active or inactive mainly relied on the counts of EOS that was subject to the pathologist’s observation and experience. Several studies have explored the value of commonly used laboratory tests as an active marker of EGPA, such as absolute eosinophil count, serum IgE, erythrocyte sedimentation rate, and C-reactive protein (CRP) (7–10). However, these tests have substantial limitations as longitudinal markers for EGPA activity (11). Recent studies revealed several novel biomarkers, such as TARC/CCL17 (12), eotaxin-3/CCL26 (13), and IgG4 (14), but their use for routine diagnosis has not yet been implemented. In our previous study, we also found that serum levels of Axl, OPN, HCC-4, GDNF, and McP-3 were consistently higher in active EGPA, independent of the assessment methods. The Axl had the highest AUC, suggesting that Axl may be a new biomarker for the diagnosis of EGPA activity (15). These findings mainly focused on classifying EGPA into active or inactive but less focused on distinguishing EGPA from severe asthma, even though the importance of differentiating between EGPA and severe asthma has been well understood (16). In addition, we used high-resolution CT (HRCT) for a clinical examination to differentiate EGPA from severe asthma in previous studies, and the results showed that HRCT was very effective (17). However, to date, molecular biomarkers for the differential diagnosis of EGPA and severe asthma are still sorely lacking and should be valuable. In addition, better diagnostic criteria at the molecular level should be established to classify different clinical and pathophysiological subtypes, which could be managed better with more specifically adapted therapies.

Nowadays, the study of biomarkers for disease diagnosis mainly uses omics research approaches, but unfortunately, few of them have been successfully translated into a Food and Drug Administration (FDA)-approved clinical test. The recent emergence of data-independent acquisition (DIA) represents a major advance in protein quantification and is significant due to its capacity to conduct high-throughput quantitative proteomics (18, 19). The parallel reaction monitoring (PRM) assay emerged as a targeted quantification mass spectrometry method with a high resolution and a high mass accuracy mode (20). Studies have shown that a novel combination of untargeted DIA and targeted PRM shows great potential in comprehensively identifying predictive candidate biomarkers for a variety of diseases such as lung adenocarcinoma (21), cancers (22), molecular typing of diseases (23), and prognostic biomarkers (24). Therefore, in this study, we will use quantitative proteomics DIA and targeted quantitative proteomic PRM to screen candidate biomarkers that can differentiate patients with asthma from patients with EGPA, so that patients with EGPA can be better managed.

## Materials and Methods

### Study Design and Collection of Clinical Samples

**Figure 1:**
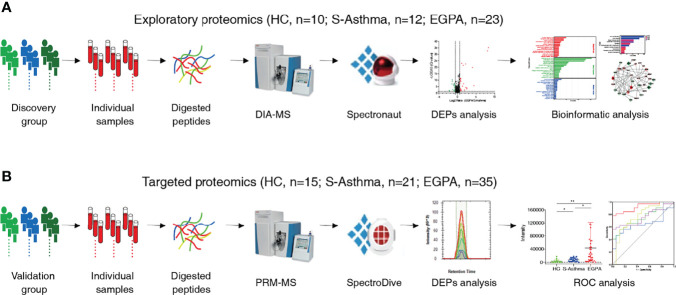
Schematic workflow of the study. Workflow for the **(A)** exploratory proteomics and **(B)** targeted proteomics.

The study design is shown in **Figure 1**. A total of 116 clinical serum samples, which were divided into the discovery group and validation group, were collected from The First Affiliated Hospital of Guangzhou Medical University from October 2016 to February 2019. Physical examination, biochemical data, and pulmonary function were obtained at the enrollment. The participants comprised 58 EGPA, 33 severe-asthma patients, and 25 healthy volunteers. The EGPA patients met the ACR classification criteria (4). Severe asthma was defined according to Global Initiative for Asthma (GINA, 2016), which fulfilled asthma that requires Step 4 or 5 treatment to prevent it from becoming “uncontrolled” or asthma that remains “uncontrolled” despite this treatment (25). Healthy volunteers had no prior history of respiratory and autoimmune disease. These subjects underwent a standardized assessment including age, body mass index, pulmonary function, and blood examinations (**Table 1**; **Supplementary Table 7**). All EGPA patients tested negative for ANCAs. Written informed consent was obtained from all patients. The study was approved by the ethics committee of the First Affiliated Hospital of Guangzhou Medical University (clinical study registration number: ChiCTR-IIC-15007622).

**Table 1:** Summary of the study cohorts.

Cohorts	Discovery group					Validation group				
Characteristics	HC (n = 10)	S-A (n = 12)	EGPA (n = 23)	*P*1 value	*P*2 value	HC (n = 15)	S-A (n = 21)	EGPA (n = 35)	*P*3 value	*P*4 value
Female, No. (%)	6 (60.00)	6 (50.00)	8 (34.78)	0.257	0.477	7 (46.67)	9 (42.86)	14 (40.00)	0.759	>0.999
Age (years)^*^	24.70 ± 1.16	46.25 ± 8.26	45.65 ± 13.97	<0.001	0.857	28.20 ± 6.24	51.33 ± 10.04	47.54 ± 13.13	<0.001	0.361
Duration of asthma (years)^*^	0	8.42 ± 6.17	6.13 ± 4.87	<0.001	0.236	0	15.38 ± 13.32	4.30 ± 4.06	<0.001	<0.001
BMI (kg/m^2^)^*^	20.04 ± 1.69	23.66 ± 3.51	23.66 ± 2.98	0.001	0.952	21.68 ± 3.45	23.77 ± 2.53	24.11 ± 3.52	0.035	0.554
FEV1 (% predicted)^*^	98.94 ± 7.13	51.77 ± 18.28	75.35 ± 16.78	<0.001	<0.001	103.70 ± 11.67	71.47 ± 20.57	75.50 ± 22.44	<0.001	0.305
FVC (% predicted)^*^	95.75 ± 6.05	76.65 ± 16.33	92.65 ± 17.35	0.630	0.016	101.40 ± 9.84	93.67 ± 19.80	95.08 ± 17.69	0.216	0.883
FEV1/FVC (%)^*^	90.73 ± 5.44	55.98 ± 13.28	68.28 ± 7.87	<0.001	0.001	89.19 ± 8.68	62.67 ± 10.27	66.69 ± 14.41	<0.001	0.259
Neutrophils (10^9^ cells/L)^*^	3.94 ± 0.35	3.85 ± 0.38	4.64 ± 1.69	0.347	0.237	3.83 ± 0.51	4.70 ± 1.35	4.96 ± 1.90	0.030	0.647
Eosinophils (10^9^ cells/L)^*^	0.14 ± 0.16	0.43 ± 0.19	0.99 ± 1.34	<0.001	0.259	0.13 ± 0.11	0.24 ± 0.21	0.82 ± 0.95	<0.001	0.002
No. of exacerbations in the past year^*^	0	3.43 ± 4.54	1.26 ± 0.81	<0.001	0.742	0	0.84 ± 1.07	1.37 ± 1.29	<0.001	0.036
Oral cs (mg/day)^*^	0	0	13.37 ± 9.70	<0.001	<0.001	0	2.38 ± 4.36	9.09 ± 7.12	<0.001	<0.001
Intranasal cs (µg/day)^*^	0	666.70 ± 105.60	790.90 ± 769.90	<0.001	0.367	0	781.00 ± 767.70	1,029.00 ± 1,034.00	<0.001	0.339
Oral cs, No. (%)	0	0	20 (86.96)	<0.001	<0.001	0	5 (23.81)	27 (77.14)	<0.001	<0.001
Intranasal cs, No. (%)	0	12 (100.00)	18 (78.26)	<0.001	0.273	0	19 (90.48)	28 (80.00)	<0.001	0.234
Organ involvement										
Lung, No. (%)	–	–	23 (100.00)	–	–	–	–	35 (100.00)	–	–
Heart, No. (%)	–	–	5 (21.74)	–	–	–	–	1 (2.86)	–	–
Nervous system, No. (%)	–	–	0	–	–	–	–	1 (2.86)	–	–
Kidney, No. (%)	–	–	1 (4.35)	–	–	–	–	1 (2.86)	–	–
Ear–nose–throat, No. (%)	–	–	0	–	–	–	–	2 (5.71)	–	–
Digestive system, No. (%)	–	–	2 (8.70)	–	–	–	–	5 (14.29)	–	–
Skin, No. (%)	–	–	6 (26.09)	–	–	–	–	4 (11.43)	–	–
BVAS score^*^	–	–	9.70 ± 2.65	–	–	–	–	8.47 ± 1.90	–	–
Median time of follow-up (months)^#^	–	6.50 (4.20, 10.00)	11.43 (6.93, 13.50)	–	0.010	–	5.17 (4.60, 9.47)	8.57 (5.33, 13.73)	–	0.016
ANCA										
Negative, No. (%)	10 (100.00)	12 (100.00)	23 (100.00)	>0.999	>0.999	15 (100.00)	21 (100.00)	35 (100.00)	>0.999	>0.999


Data expressed as mean ± SD (^*^), median (interquartile range, ^#^), or number (No., %). The p-value was calculated from the Mann–Whitney t-test between groups. P1 and P3: the p-value between EGPA and HC groups based on discovery or validation group, respectively. P2 and P4: the p-value between EGPA and S-A groups based on discovery or validation group, respectively. BMI, body mass index; FEV1, forced expiratory flow in 1 s; FVC, forced vital capacity; cs, corticosteroid; BVAS, Birmingham Vasculitis Activity Score; ANCA, anti-neutrophil cytoplasmic antibodies.

For serum collection, blood samples were collected into serum separator tubes, allowed to clot for 1 h at room temperature, then centrifuged at 1,000 *g* for 10 min at 4°C, and then stored in aliquots at −80°C. Meanwhile, 10 µl of serum from each sample was mixed as a pooled sample, which was used as a QC sample and data-dependent acquisition (DDA) analysis.

### Sample Preparation

The protein digestion was performed by filter-aided sample preparation (FASP) (26). First, each aliquot of 200 µg of depleted proteins was then diluted to 200 µl with 8 M of UA buffer (8 M of urea in 0.1 M of Tris-HCl, pH 8.5). The sample solution was centrifuged on a 10-kDa filter (OD010C34; PALL, Port Washington, NY, USA) for 20 min. Then, a mixture of 200 µl of 8 M UA buffer and 200 µl of 10 mM dithiothreitol (DTT) (D8220; Solarbio, Beijing, China) was added, and the reduction reaction was kept for 1 h at room temperature. The solution was removed by centrifugation for 20 min, and 200 µl of UA buffer with 200 µl of 50 mM iodoacetamide (IAA) (I6125; Merck, Kenilworth, NJ, USA) was added and then was incubated in the dark for 30 min at room temperature. The ultra-fraction tube was washed with 300 µl of ABC (50 mM of ammonium bicarbonate) three times by centrifugation at 12,000 *g* for 20 min at room temperature. Then, 40 µl of ABC containing 0.1 µg/µl of trypsin (V5280; Promega, Madison, WI, USA) was added to each filter tube and then was incubated at 37°C overnight. The peptides were collected into a low-binding collection tube (88379; Thermo Scientific™, USA) by centrifugation at 12,000 *g* for 20 min. The filter tubes were washed twice with 50 µl of ABC by centrifugation at 12,000 *g* for 20 min. The flow-through peptides were collected and pooled, and then the concentration was measured using Pierce Quantitative Fluorometric Peptide Assay (23290; Thermo Scientific™, Waltham, MA, USA). The peptide mixtures were desalted on Waters C18 columns (WAT054955; Waters, Milford, MA, USA) using a 20-Position Extraction Manifold. In brief, the C18 columns were conditioned with 1 ml of acetonitrile (ACN) (A955-4; Thermo Scientific™, USA) and 1 ml of ultrapure water and then equilibrated with 1 ml of 5% ACN with 0.5% trifluoroacetic acid (TFA) (T818782; Macklin, Shanghai, China). Peptide mixtures measuring 150 μg, which were acidized to pH 3–4 with 15 µl of 2% TFA, were loaded onto the C18 resin bed, and then the C18 resin was washed with 1 ml of 5% ACN with 0.5% TFA. Then the purified peptide mixtures were eluted with 70% ACN and collected into low-binding tubes, dried by vacuum centrifugation, and then stored at −80°C.

### Data-Dependent Acquisition Datasets

For DDA analysis, high-abundance protein depletion and peptide pre-fractionation were carried out for the pooled serum samples. The purified peptide mixtures were fractionated using high-pH reversed-phase liquid chromatography (RPLC) (27). In brief, 500 µg peptide mixture was re-dissolved in 500 µl of 2% (v/v) ACN in water (buffer A, pH 10) and loaded onto the C18 column (4.6 × 250 mm, C18, 3 µm, 186003581; Waters, USA) in buffer A. The elution gradient was 5%−45% buffer B (98% ACN, pH 10; flow rate, 1 ml/min) for 60 min, and the eluted peptides were collected one fraction per minute and finally merged into 15 fractions by combining fractions 1, 15, 30, 45, and so on. Fractionated peptides were dried by vacuum centrifugation and reconstituted in 0.1% (V/V) formic acid (FA) (A117-50; Thermo Scientific™, USA) in water to a final concentration of 0.5 µg/µl, and each of them was divided into 3 aliquots for 3 replicates using DDA approach. For DDA analysis, shotgun proteomics was performed using a Q Exactive mass spectrometer equipped with EASY-nLC 1000 system (Thermo Fisher Scientific, USA) operating in the DDA mode. Of each of the fractions containing 0.2 µl of standard peptides (iRT kit) (Ki-3002-2; Biognosys, Schlieren, Switzerland), 2 µl was loaded on a nano trap column (Acclaim PepMap100 C18, 100 µm × 20 mm, 5 μm, AAA-164564; Thermo Scientific™, USA) and then separated onto an analytical column (PepMap C18, 75 μm × 250 mm, 2 μm, 164941; Thermo Scientific™, USA) using a 120-min linear gradient (solvent A: 98% H_2_O, 2% ACN, 0.1% FA; solvent B: 98% ACN, 2% H_2_O, 0.1% FA) at a flow rate of 300 nl/min. The detailed solvent gradient was as follows: 3%–7% B, 4 min; 7%–18% B, 70 min; 18%–25% B, 20 min; 25%–35% B, 16 min; 35%–40% B, 1 min; and 40%–90% B, 9 min. The mass spectrometer was operated in data-dependent top 20 modes with the following settings: MS1 scan was acquired from 400 to 1,200 *m*/*z* with a resolution of 70,000, the auto gain control (AGC) was set to 3e6, and the maximum injection time was set to 60 ms. MS2 scans were performed at a resolution of 17,500 with an isolation window of 1.6 *m*/*z* and higher-energy collision dissociation (HCD) at 32%, the AGC target was set to 5e5, the maximal injection time was 50 ms, the loop count was set to 20, and the normalized collision energy (NCE) was 27%, with dynamic exclusion of 30 s. The MS raw data for DDA are publicly available in iProX (accession number: IPX0003689001).

### Data-Independent Acquisition Datasets

For DIA analysis, 1 µg of digested peptides containing 0.2 µl of standard peptides of each sample was analyzed in the DIA method. Each sample was injected once; to control for technical variables, the QC sample, which was pooled from each serum sample, was analyzed every ten samples. The liquid conditions were the same as those of the DDA model. For MS acquisition, the MS1 resolution was 70,000, and the MS2 resolution was set to 17,500. The *m*/*z* range covered from 400 to 1,200 *m*/*z* and was separated into 30 variable acquisition windows (**Supplementary Table 1**). The full-scan AGC target was set to 3e6, with an injection time of 60 ms. DIA settings included NCE of 27%, AGC target of 1e6, and auto maximum injection time. The MS raw data for DIA are publicly available in iProX (accession number: IPX0003689001).

### Liquid Chromatography–Mass Spectrometry Data-Independent Acquisition Data Analyses

The generation of an appropriate spectral library containing targeted MS information of interested proteins is important for DIA experiments (28). For the generation of a comprehensive spectral library, DDA raw data of 15 fractions and DIA raw data of 45 serum samples were processed using Spectronaut Pulsar X (Biognosys, Switzerland) with default settings (**Supplementary Figure 1**). The search allowed 2 missed cleavages, and the enzyme was set to trypsin/P. Carbamidomethyl (cysteine) was allowed as a fixed modification, and oxidation (methionine) and acetyl (protein N-term) were allowed as variable modifications. The database was Uniprot-Human-Filtered-Reviewed-Yes 191007. fasta. The mass tolerance for matching precursor and fragment ions was dynamic. The identification was performed using a 0.01 false discovery rate (FDR) threshold on the peptide, protein, and peptide-spectrum match (PSM). The DDA and DIA raw data were then imported to Spectronaut Pulsar to generate spectral libraries, and then libraries could be merged by selecting the “Merge” option.

DIA data were analyzed with Spectronaut Pulsar X based on the merged spectral library using the default settings. The calibration was set to non-linear iRT calibration with precision iRT enabled. The identification was performed using a 0.01 Q-value (adjust *p*-value) cutoff on precursor and protein levels, while the maximum number of decoys was set to a fraction of 0.75 of library size. For quantification, interference correction was enabled with at least three fragment ions used per peptide; the major and minor group quantities were set to mean peptide and mean precursor quantity, respectively, with the top 3 group selection each. Quantity was determined on the MS2 level using the area of XIC peaks with enabled cross-run normalization.

### Parallel Reaction Monitoring Analyses

To yield a reliable quantification of the selected proteins, a criterion for selecting target peptides was included, as follows: the missed cleavage was set to 0, and 1–3 unique peptides were selected for each protein. The peptide length was filtered by 8–25 amino acids, and doubly or triply charged precursor ions were selected (29). At least one best flying peptide was selected for each protein, and 3–6 transitions were considered for each peptide. After the peptides were selected, the information of the target peptides including *m*/*z*, charge number, and charge type was input into the “inclusion list.” The mixed peptides described above were analyzed by a “full scan” followed by a “PRM” pattern.

The serum pooled sample was prepared as a QC sample for PRM unscheduled data acquisition. Of the 62 serum samples containing 0.2 µl of standard peptides, 1 μg of digested peptides was analyzed using PRM scheduled acquisition (**Supplementary Table 2**), which is based on the unscheduled PRM analysis. To assess the reproducibility and technical variables, each sample was injected three times. Furthermore, a pooled QC sample was injected every four batches. PRM analysis was performed on a Q Exactive mass spectrometer equipped with EASY-nLC 1000 system with a 60-min gradient as follows: 3%–7% B, 1 min; 7%–22% B, 35 min; 22%–30% B, 11 min; 30%–80% 8 min; and 80%–90% B, 5 min. For MS acquisition, the MS1 resolution was 70,000, with *m*/*z* ranging from 350 to 1,800 *m*/*z*, and the AGC target was set to 3e6. The MS2 resolution was set to 17,500, and the AGC target was set to 2e5, with an injection time of 50 ms, the loop count was set to 20, and the NCE was 27%, with an isolation window of 1.6 *m*/*z*. PRM raw data were analyzed by Spectrodive (Biognosys, Switzerland) using the default settings. Peptides were quantified by summing the peak areas under the curve (AUCs) of each transition, and the mean of best flying peptides was used to measure the abundance of proteins. The MS raw data for PRM are publicly available in iProX (accession number: IPX0003689001).

### Statistical and Bioinformatics Analyses

Multiple t-test analyses with a two-stage linear step-up procedure of Benjamini, Krieger, and Yekutieli to determine FDR < 0.05 were used for the detection of differentially abundant proteins across the different groups (biomarker discovery study). To analyze the changes in the clinical and hematological variables between the different groups, as well as for PRM results, the Kruskal–Wallis one-way ANOVA followed by Dunn’s tests was performed. The non-parametric Spearman’s correlation test was performed to analyze the associations between the serum candidate biomarkers and blood eosinophil count. All of these analyses were carried out with GraphPad Prism 9.0. The median time of follow-up and its interquartile range were carried out with SPSS 20.0 according to the reverse Kaplan–Meier method. Receiver operating characteristic (ROC) curves and AUCs, which were carried out with SPSS 20.0 software, were used to evaluate the discriminatory power of the candidate biomarkers. The pair of sensitivity and specificity that correspond to the maximum of the Youden index (YD) were calculated to characterize the performance of the candidate biomarkers. A *p*-value of <0.05 was considered significant. Principal component analysis (PCA), Gene Ontology (GO) annotation, and Kyoto Encyclopedia of Genes and Genomes (KEGG) analysis were performed using an omicsolution web-based platform version 34.0 (http://wkomics.omicsolution.com/wkomics/main/) (30). PCA was carried out using a statistical analysis module with default parameters, and GO and KEGG analyses were carried out using a functional analysis module with enriched top 15 and top 10 items, respectively. Protein–protein interaction (PPI) analyses were performed using the Search Tool for the Retrieval of Interacting Genes (STRING) and Cytoscape 3.9.0.

## Results

### Identification of Differentially Expressed Proteins

DIA is a new label-free quantitative technique in proteomics that has emerged in recent years. It is a powerful screening technique for the comprehensive and reproducible quantitation of biological samples. As the method sequentially collects MS/MS fragmentation spectra on all ions within a given *m*/*z* range, it affords the opportunity for retrospective analysis of unknowns and new targets of interest. Therefore, DIA mass spectrometry was selected as the worth-watching technology in the coming years in 2015 by *Nature Methods* (31). At present, there are several mainstream methods for quantitative analysis of DIA data, including 1) quantitative analysis based on spectrum library established by DDA data (32–34); 2) quantitative analysis was carried out based on the spectrum library established by DIA data (32, 35, 36); 3) Direct_DIA (32, 37, 38); and 4) quantitative analysis was carried out based on the spectrum library established by combining DDA and DIA data (32, 37, 39). For the first method, the advantage is high protein quantification accuracy, but the disadvantage is low proteome coverage. In the second approach, DIA data can be processed and searched directly in the FASTA sequence database; however, this library-free approach generally results in lower proteome coverage. The Direct_DIA method is based on the independent prediction of fragment ion intensity and peptide retention time in the deep learning model to construct a virtual spectral library for data searching analysis. This method can almost achieve full proteome coverage. However, this would result in the substantial expansion of the spectral library and the resulting significant increase in the FDR. The fourth method is to build a hybrid spectrum library combining DDA and DIA data. This method is a supplement to the previous three methods and has the advantages of other methods while effectively avoiding the shortcomings of the other three methods. Thus, an appropriate spectral library such as a sample-type-specific spectral library is beneficial for reliable DIA identification and high reproducibility (32, 37, 39). Therefore, to obtain more comprehensive data, we performed RPLC pre-fractionation of pool serum samples before collecting DDA data and identified a total of 1664 proteins through three biological replicates (**Supplementary Figure 2A**). Subsequently, we tested the application of the above four methods in our sample species and found that the quantitative method based on the spectrum library established by combining DDA and DIA data could quantify the maximum number of proteins (**Supplementary Figures 2B, C**). Therefore, the subsequent DIA quantitative analysis in this study was based on the spectrum library established by combining DDA and DIA data.

**Figure 2:**
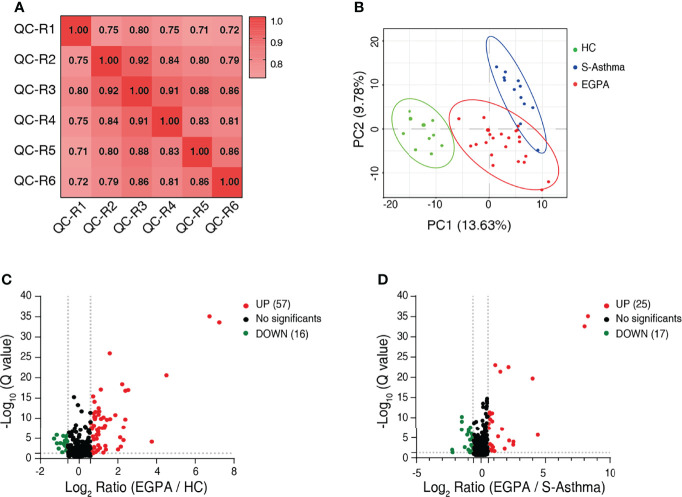
Identification of differentially expressed proteins. **(A)** Correlation analysis to validate the technical reproducibility of quality control sample analysis using the DIA approach. **(B)** PCA score plot of the serum samples of discovery cohort showing clear separation of healthy controls from severe-asthma and EGPA patients. **(C)** Volcano plot shows the DEPs between EGPA and healthy control groups. **(D)** Volcano plot shows the DEPs between EGPA and severe-asthma groups. Green dots indicate downregulated proteins, red dots indicate upregulated proteins, and the black dots indicate the proteins with no significant difference. DIA, data-independent acquisition; PCA, principal component analysis; EGPA, eosinophilic granulomatosis with polyangiitis; DEPs, differentially expressed proteins.

To discover potential biomarkers effectively and carry out validation, we divided the study cohort into the discovery group and validation group (**Figure 1**). In the discovery cohort of 45 serum samples, we performed quantitative proteomic analysis using the DIA method to identify candidate biomarkers for EGPA (**Figure 1A**). During the DIA data acquisition process, we used the pool serum samples as QC criteria and collected data six times at different time points to evaluate the technical reproducibility of the DIA method. Our results showed that the total ion chromatogram (TIC) was evenly distributed within the gradient range, the peak time was relatively stable (**Supplementary Figure 3A**), and the full peak width at half maximum of each peptide segment was about 0.3 (**Supplementary Figure 3B**). These results indicated that the chromatographic column performance of our instrument was stable. In addition, we also used the standard peptide iRT as correction for DIA data collection, and the results showed that the detection of the iRT peptide was stable among all samples (**Supplementary Figure 3C**), indicating that the protein quantification of samples in this experiment was of high accuracy. The analysis results of QC samples also showed that the quantitative correlation coefficient R-value of the data collected at six different time points was above 0.71 (**Figure 2A**), indicating that the mass spectrometry data collection of this project was quite stable. PCA of all samples showed that the healthy control group, EGPA group, and asthma group were well separated, which not only reflected the specificity of each disease but also illustrated the diagnostic accuracy of our samples (**Figure 2B**). When analyzing differentially expressed proteins (DEPs), we found that the EGPA group had a total of 73 DEPs including 57 upregulated proteins and 16 downregulated proteins, compared with healthy controls (**Figure 2C**; **Supplementary Table 3**). There were 42 DEPs in the EGPA group compared with the asthma group, including 25 upregulated proteins and 17 downregulated proteins (**Figure 2D**; **Supplementary Table 4**). These data suggest the potential for finding suitable diagnostic biomarkers for early diagnosis of EGPA and for differentiating asthma in our study cohort.

### Identification of Candidate Markers

**Figure 3:**
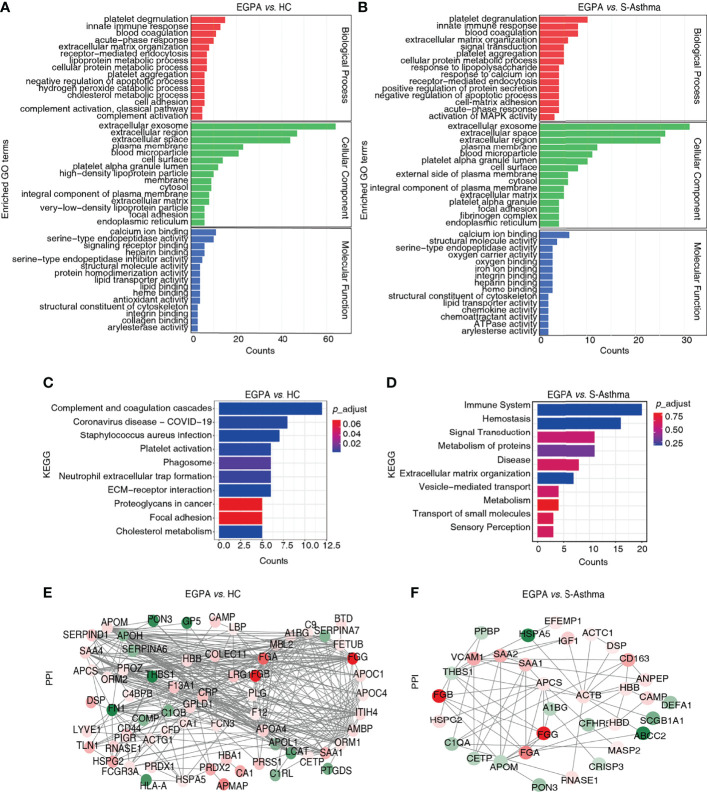
Bioinformatics analysis of the DEPs. **(A, B)** GO classification of the DEPs. The top 15 enriched terms in the Biological Process (BP), Cellular Component (CC), and Molecular Function (MF) are listed. **(C, D)** KEGG pathway analysis of the DEPs. The top 10 enriched pathways are listed. **(E, F)** PPI network of the DEPs. DEPs, differentially expressed proteins; GO, Gene Ontology; KEGG, Kyoto Encyclopedia of Genes and Genomes; PPI, protein–protein interaction.

Subsequently, we analyzed the DEPs by bioinformatics methods with the aim of screening candidate biomarkers that can characterize EGPA disease. GO analysis results showed that the DEPs were mainly distributed in extracellular and cell membrane regions and were mainly involved in biological processes such as platelet degranulation, innate immune response, and blood coagulation, in the EGPA group compared to the healthy control or the asthma group. The binding of cofactors was the most significant molecular function of the DEPs (**Figures 3A, B**). In addition, the KEGG analysis suggested that these DEPs were mainly involved in the immune system and hemostasis (**Figures 3C, D**). The PPI analysis yielded a highly clustered network (clustering coefficient = 0.47 and enrichment *p*-value < 0.05) enriching in platelet degranulation, which is strongly associated with vasculitis properties of EGPA (**Figures 3E, F**). Based on these results, 23 DEPs were selected as the candidate biomarkers for further PRM analysis (**Supplementary Table 5**).

### Validation and Evaluation of Candidate Biomarkers: SAA1, FGA, SAP, and CETP

**Figure 4:**
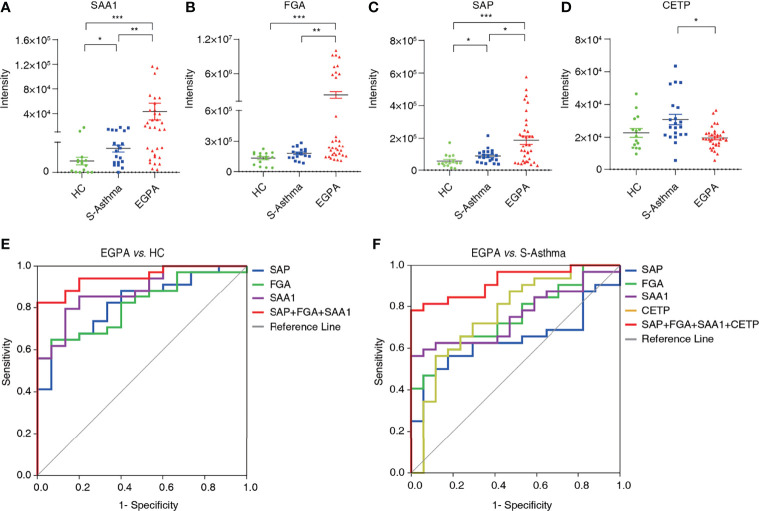
Validation and evaluation of candidate biomarkers. The quantification of serum SAA1 **(A)**, FGA **(B)**, SAP **(C)**, and CETP **(D)** in healthy control, severe asthma, and EGPA groups (*p < 0.05; **p < 0.01; ***p < 0.001). **(E)** Receiver operating characteristic curve analysis of candidate biomarkers for EGPA vs. HC. **(F)** Receiver operating characteristic curve analysis of candidate biomarkers for EGPA vs. S-asthma. EGPA, eosinophilic granulomatosis with polyangiitis; HC, healthy controls.

We then validated the candidate biomarkers using PRM. Four proteins including SAA1, FGA, SAP, and CETP observed good consistency between DIA and PRM results, indicating that our detection results are reliable (**Supplementary Table 6**). The serum levels of SAA1, FGA, and SAP in the EGPA group were significantly elevated as compared to the healthy control and severe-asthma groups (**Figures 4A–C**), while the serum CETP was significantly lower in the EGPA group compared with the severe-asthma group (**Figure 4D**). It has been reported that serum SAA protein in EGPA patients is significantly higher than that in healthy subjects (40). The other three, FGA, SAP, and CETP, have not been reported as EGPA disease-related biomarkers so far, suggesting that we may have found new diagnostic biomarkers for EGPA.

ROC analysis was used to evaluate the efficacy of candidate biomarkers. We found that EGPA *vs.* HC, SAA1, FGA, and SAP alone had AUC scores above 0.8, sensitivity scores above 60%, and specificity scores above 85%, showing good clinical value. However, when SAA1, FGA, and SAP were combined, the AUC, sensitivity, and specificity scores were greatly improved, and the specificity even reached 100%. These results suggest that the combination of SAA1, FGA, and SAP can be used as biomarkers for the early diagnosis of EGPA. In the EGPA *vs.* asthma analysis, SAA1, FGA, SAP, and CEPT alone had slightly lower sensitivity but still showed good AUC and specificity. When the four subjects were combined, the AUC score was 92.1% and the specificity score was 100%, indicating a good clinical evaluation (**Figures 4E, F**; **Table 2**). These results suggest that the combination of SAA1, FGA, SAP, and CEPT can be used as biomarkers in the differential diagnosis of EGPA and asthma.

**Table 2:** Diagnostic value of candidate biomarkers for distinguishing EGPA from healthy control and severe-asthma groups.

	EGPA vs. HC			EGPA vs. S-asthma		
Items	AUC (95% CI)	Sen. %	Spe. %	AUC (95% CI)	Sen. %	Spe. %
SAA1	0.880 (0.785–0.976)	79.41%	86.67%	0.756 (0.623–0.888)	56.25%	100.00%
FGA	0.814 (0.696–0.931)	64.71%	93.33%	0.750 (0.616–0.884)	56.25%	88.24%
SAP	0.824 (0.706–0.941)	64.71%	93.33%	0.642 (0.488–0.795)	46.88%	94.12%
CETP	–	–	–	0.765 (0.617–0.912)	56.25%	88.24%
Combination	0.947 (0.890–1.000)	82.35%	100.00%	0.921 (0.848–0.994)	78.13%	100.00%

AUC, area under the curve; Sen., sensitivity; Spe., specificity; EGPA, eosinophilic granulomatosis with polyangiitis; HC, healthy controls.

### The Correlation Between SAA1, FGA, SAP, CETP, and Eosinophil Count

**Figure 5:**
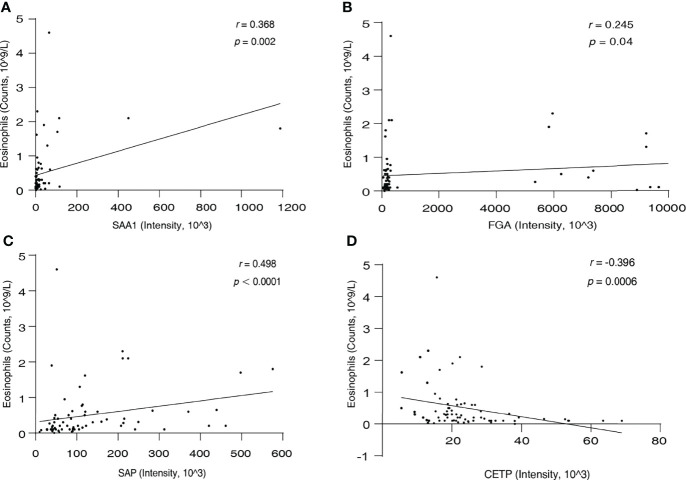
The correlation between serum SAA1 **(A)**, FGA **(B)**, SAP **(C)**, CETP **(D)**, and eosinophil count.

Eosinophils are the most characteristic cells in EGPA. Therefore, we next performed a correlation analysis between the serum levels of the candidate biomarkers and blood eosinophil count in all participants. Result as shown in **Figure 5**; SAA1 (*r* = 0.368, *p* = 0.002), FGA (*r* = 0.245, *p* = 0.04), and SAP (*r* = 0.498, *p* < 0.0001) were positively correlated with eosinophil count, and CETP (*r* = −0.396, *p* = 0.0006) was negatively correlated with eosinophil count. These results suggest that the four new biomarkers can well characterize EGPA disease and are significantly associated with the eosinophilic inflammation of EGPA.

## Discussion

Due to the lack of an effective diagnostic biomarker, the majority of EGPA patients were usually misdiagnosed with severe asthma. In particular, 98% of EGPA patients are misdiagnosed with severe asthma in the first stage of EGPA development, which is distinguished by the occurrence of asthma, allergic rhinitis, and sinusitis. These patients were treated with inhaled corticosteroids (ICS) to achieve remission induction. However, this treatment is not suitable for EGPA and may cover up the clinical features, such as eosinophilic infiltrations and vascular symptoms, resulting in delayed treatment, reduced quality of life, and even disability. Therefore, extensive studies are still needed to find more valuable biomarkers to distinguish EGPA from severe asthma. In this study, we used a DIA-based quantitative proteomics approach to investigate the potential candidate biomarkers for EGPA diagnosis in serum. There were 42 significantly DEPs discovered between the EGPA and severe-asthma groups. Bioinformatics analysis of these DEPs suggested that they were mainly located in the extracellular region and membrane parts, presented with binding function in the processes of platelet degranulation, innate immune response, and blood coagulation. Then, a total of 23 candidate biomarkers were selected to validate the different levels in serum samples *via* the PRM approach. Among them, 4 proteins, including SAA1, FGA, SAP, and CETP, exhibited similar upward or downward trends as observed with the DIA approach.

SAA1 (serum amyloid A1) is well known as a hallmark of the acute-phase response. Accumulating evidence indicates that elevated serum levels of SAA can be a clinical biomarker for inflammatory diseases (41). A recent study has shown that SAA levels in serum of EGPA patients were higher than in healthy controls by using the ELISA method, and it has suggested that SAA may reflect the process of fibrogenesis after the granulomatous process (40). Both DIA and PRM approaches applied in our study showed that serum SAA1 level in the EGPA group was not only significantly higher than that in the healthy control group, which was consistent with the previous study, but also significantly higher than the severe-asthma group. These results suggest that SAA1 may be a biomarker not only for early diagnosis of EGPA but also for differential diagnosis of asthma. Therefore, it is worthwhile to further investigate the potential molecular mechanism of EGPA involvement.

Fibrinogen-α (FGA), a major plasma protein coagulation factor, is a soluble glycoprotein primarily synthesized in the liver by hepatocytes and is also a major acute-phase reactant. The synthesis of plasma fibrinogen is upregulated in response to inflammatory mediators such as IL-6 (42). Therefore, it was plausibly considered a non-invasive measurement of ongoing airway inflammation and lung tissue destruction. Elevated fibrinogen levels have been observed in subjects with several chronic lung diseases including chronic obstructive pulmonary disease (COPD), which have inflammation as an underlying component (43, 44). However, it was not been reported in EGPA disease-related studies. For the first time, serum fibrinogen-α, as well as fibrinogen-β and fibrinogen-γ, was observed significantly increased in the EGPA patient group compared with the healthy controls and severe-asthma group in the present study. Fibrinogen, the thrombin substrate that produces fibrin, is known to play a critical role in controlling bleeding upon vascular injury. It is also a major determinant of wound healing, tissue regeneration, and mediation of inflammatory responses and helps the immune system fight invading pathogens. On the other hand, it has been reported that fibrinogen may also play a leading role in fibrotic and arthritic diseases (45). Therefore, FGA may have important guiding significance as a new biomarker for the early diagnosis of EGPA and differential diagnosis of asthma.

SAP (serum amyloid P component, also known as PTX2) is a member of the pentraxin family, which includes CRP (PTX1) and pentraxin-3 (PTX3). It plays an important role in the regulation of the innate immune system, with various functions such as decreasing neutrophils adhesion (46), inhibiting fibrocyte differentiation (47), and regulating macrophage activation (48). As we all know, CRP as an acute-phase protein is widely used in the diagnosis and treatment of autoimmune diseases or infections (49). It has been reported that the level of CRP was associated with EGPA, but several analyses showed that the CRP test lacks adequate sensitivity for EGPA diagnosis (11). In our study, DIA analysis showed no statistically significant difference in CRP levels between the EGPA and the severe-asthma groups. However, PRM analysis results showed that the CRP level in the EGPA group was significantly higher than that in the severe-asthma group. Therefore, the sample size may be increased for further verification and confirmation. PTX3, another member of the pentraxin family, has been reported to be involved in various systemic immune-mediated diseases. It has been reported that vascular inflammation was the main driver of PTX3 elevation (50). In this study, both DIA analysis and PRM analysis showed that the SAP level in the EGPA group was significantly higher than that in the severe-asthma group. These results suggest that pentraxin family proteins, especially SAP, may be involved in the pathogenesis of EGPA, but the biological mechanisms need to be further studied.

In our study, we found that serum CETP levels in patients with the EGPA group were significantly lower than those in the severe-asthma group. A previous study showed that hydrogen peroxide level is related to CETP expression, and CETP is involved with increased vascular reactive oxygen species (ROS) (51). In the pathogenesis of EGPA, the presence of ANCA is an important factor in the occurrence and development of EGPA. ANCA binds to activated neutrophils *via* Fcγ receptors or MPO protein expressed on the cell surface, resulting in ROS production and the release of proteolytic enzymes, forming neutrophil extracellular traps (NETs) (1). Therefore, CETP may play an important role in the pathogenesis of ANCA-mediated EGPA. However, all the EGPA subjects we included were ANCA negative, and the result of decreased CETP levels in the EGPA group compared with the severe-asthma group suggests that the application of CETP may be useful for discriminating ANCA positive or negative EGPA. Collectively, these results confirm that EGPA is a puzzling disease and commonly consists of a combination of asthma, granulomatous, and vasculitis, accompanied by EOS-rich inflammation. Further, investigation on EGPA reveals that inflammation is associated with the development and progression of the disease, and inflammatory factors may be applied as diagnostic indicators for EGPA.

Eosinophils are the major cells responsible for EGPA. The critical role of eosinophils in EGPA is well established, as demonstrated by the clinical benefit of eosinophil-targeted anti-interleukin-5 (anti-IL-5) antibody therapy (52, 53). In our study, we found that the serum SAA1, FGA SAP, and CETP were significantly correlated with blood eosinophil counts. These results might be related to the eosinophilic inflammation involved in the development of EGPA. However, the function of these four proteins and the correlation with EGPA need further confirmation.

In conclusion, our results suggest that DIA combined with PRM mass spectrometry can effectively identify and validate the candidate biomarkers for various diseases, including EGPA. We have identified and validated four new potential serum biomarkers, including SAA1, FGA, SAP, and CETP, that can be used to distinguish EGPA from severe asthma. These new potential biomarkers may have important implications for the diagnosis of EGPA. However, the diagnostic value of these potential biomarkers should be validated by other methods, such as ELISA, in a larger cohort of patients. Moreover, serial samples of patients should be divided into two groups according to the active stage and inactive stage, and appropriate biomarkers should be further searched to distinguish the active stage and inactive stage of EGPA, so as to better manage EGPA.

## Data Availability Statement

The datasets presented in this study can be found in online repositories. The names of the repository/repositories and accession number(s) can be found in the materials and methods.

## Ethics Statement

The studies involving human participants were reviewed and approved by The First Affiliated Hospital of Guangzhou Medical University (Clinical study registration number: ChiCTR-IIC-15007622). The patients/participants provided their written informed consent to participate in this study.

## Author Contributions

FL, TP, and QZ conceived and supervised the study. JX and XW performed all the experiments with assistance from ML. FL, JX, SL, and XW performed the data mining. CD, XXZ, MQ, and CO recruited patients and collected clinical data. JX and SL wrote the manuscript, and SL contributed to critical editing. XZ contributed to the literature review and reference. YL contributed to the management and uploading of MS data to public databases. LH and LT provided guidance and suggestions for the project. All authors listed have made a substantial, direct, and intellectual contribution to the work and approved it for publication.

## Funding

This work was supported by the National Natural and Science Foundation of China (Project No. 82002949); Open Project of State Key Laboratory of Respiratory Disease (Project No. SKLRD-OP-202010); Medical Scientific Research Foundation of Guangdong Province (Project No. A2021231); Project 111 (Project No. D18010); State Key Laboratory of Respiratory Disease, Guangdong-Hong Kong-Macao Joint Laboratory of Respiratory Infectious Disease (Project No. GHMJLRID-Z-202103); Guangzhou Institute of Respiratory Health Open Project (Funds provided by China Evergrande Group, Project No. 2020GIRHHMS01); National Natural Science Foundation of China (Project No. 82070026), Zhongnanshan Medical Foundation of Guangdong Province (Project No. ZNSA-2020013, ZNSA-2020003); Natural Science Foundation of Guangdong Province (Project No. 2019A1515010622); and Guangzhou Medical University Discipline Construction Funds (Basic Medicine) (Project No. JCXKJS2022A11).

## Conflict of Interest

Author TP is employed by Guangdong South China Vaccine Co., Ltd.

The remaining authors declare that the research was conducted in the absence of any commercial or financial relationships that could be construed as a potential conflict of interest.

## Publisher’s Note

All claims expressed in this article are solely those of the authors and do not necessarily represent those of their affiliated organizations, or those of the publisher, the editors and the reviewers. Any product that may be evaluated in this article, or claim that may be made by its manufacturer, is not guaranteed or endorsed by the publisher.
