# Supplementary material for: Serum Proteomic Analysis Identifies SAA1, FGA, SAP, and CETP as New Biomarkers for Eosinophilic Granulomatosis With Polyangiitis
Source: Front Immunol. 2022 Jun 10;13:866035. doi: 10.3389/fimmu.2022.866035 (PMC9226334; doi:10.3389/fimmu.2022.866035)
Supplement: Supplementary file 8 [file Presentation_1.pdf]

Supplementary Material

1 Supplementary Figures

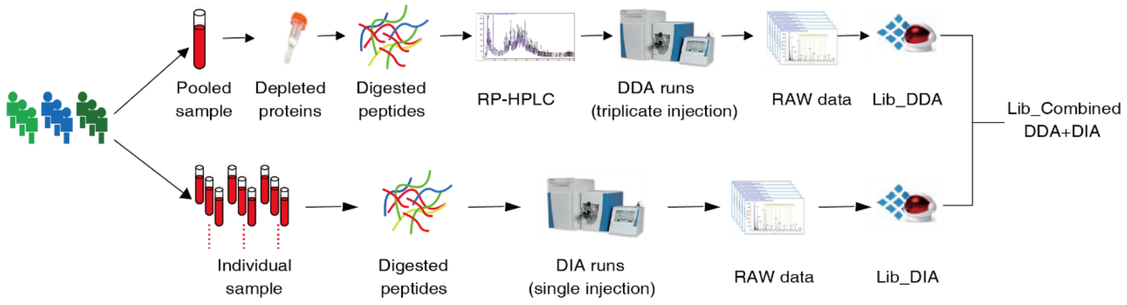

Supplementary Figure 1. Schematic workflow of spectral library construction.

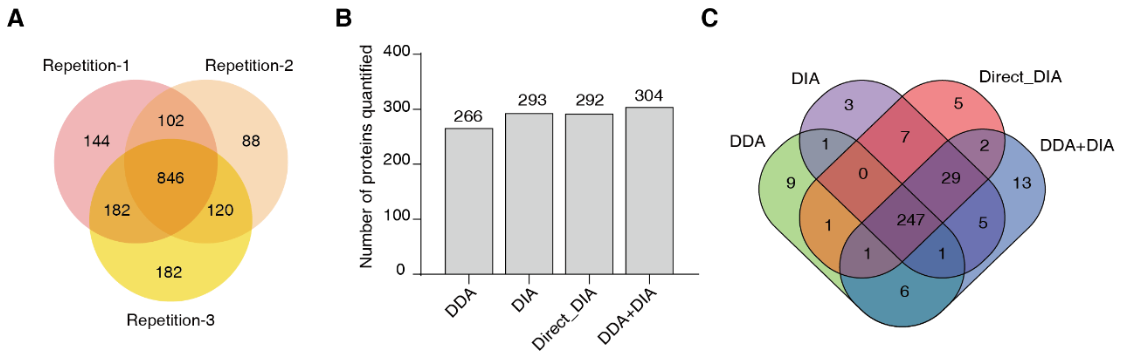

Supplementary Figure 2. Comparison of the quantified proteins according to DIA analysis using spectral libraries based on DDA, DIA, Direct_DIA and combined DDA and DIA approach. **(A)** Proteins identified by DDA analysis (three biological replicates). (**B**) Comparison of the number of quantified proteins. (**C**) Venn diagram of the quantified proteins.

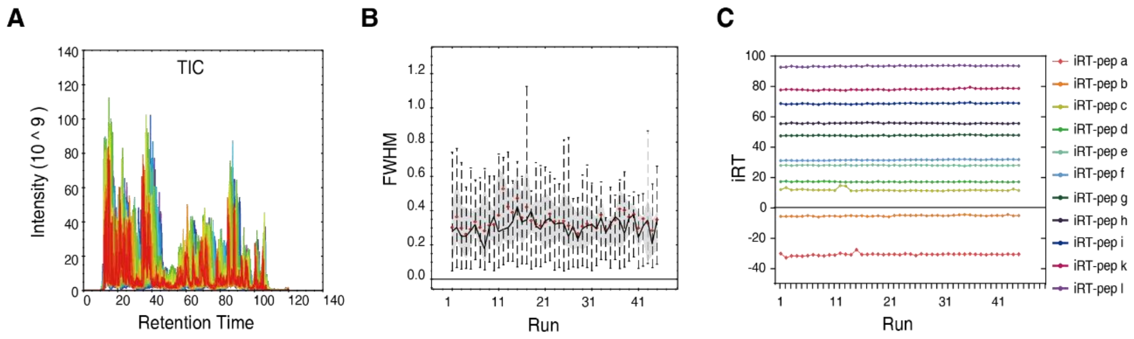

Supplementary Figure 3. Quality control of DIA analysis. **(A)** Total ion chromatogram (TIC) was evenly distributed within the gradient range, and the peak time was relatively stable. **(B)** The full peak width at half maximum (FWHM) per peak was around 0.3, which meant a stability column performance. **(C)** Chart of iRT elution time between DIA runs. The main iRTs were detected and the retention time was generally stable.

2 Supplementary Tables

Supplementary Table 1. The 30 variable acquisition windows for DIA-MS mode.
Separate excel file.

Supplementary Table 2. The scheduled method of targeted peptides for the PRM-MS method.
Separate excel file.

Supplementary Table 3. The differentially expressed proteins between EGPA and healthy control groups.
Separate excel file.

Supplementary Table 4. The differentially expressed proteins between EGPA and severe-asthma groups.
Separate excel file.

Supplementary Table 5. 23 candidate biomarkers for further PRM analysis.
Separate excel file.

Supplementary Table 6. Performance of candidate biomarkers in DIA and PRM.
Separate excel file.

Supplementary Table 7. The detailed information of the study cohort.
Separate excel file.
